# Impacts of continuing education for health professionals in primary health care: A scoping review

**DOI:** 10.1371/journal.pone.0339980

**Published:** 2025-12-31

**Authors:** Laianny Krizia Maia Pereira, José Adailton da Silva, Eva Emanuela L. Cavalcante Feitosa, Alexandre R. Caitano, Janaína Luana R. da Silva Valentim, Manoel Honorio Romão, Natalia Araújo do N. Batista, Lyane Ramalho Cortez, Karilany Dantas Coutinho, Aline de Pinho Dias, Thaísa G. F. M. S. Lima, Ricardo A. de M. Valentim, Tatyana Souza Rosendo

**Affiliations:** 1 Postgraduate Program in Family Health (RENASF), Federal University of Rio Grande do Norte, Rio Grande do Norte, Brazil; 2 Laboratory for Technological Innovation in Health (LAIS), Federal University of Rio Grande do Norte, Natal, Rio Grande do Norte, Brazil; 3 Department of Biomedical Engineering, Federal University of Rio Grande do Norte, Natal, Rio Grande do Norte, Brazil; Universidade Europeia, Lisboa, PORTUGAL

## Abstract

**Introduction:**

Continuing Health Education (CHE) is an essential strategy for the continuous qualification of Primary Health Care (PHC) professionals, promoting knowledge updating and improving the quality of services. In Brazil, this process is especially relevant given the challenges faced by the Unified Health System (SUS) and the need for training aligned with local demands. Understanding the impact of CHE actions in PHC is crucial to identify their effects on professional practice and health outcomes.

**Objective:**

To identify and analyze the evidence on the impacts of CHE for health professionals in the context of Primary Health Care in Brazil.

**Method:**

This is a scoping review conducted according to the Joanna Briggs Institute (JBI) guidelines and reported based on the PRISMA-ScR checklist. The search was performed in MEDLINE/PubMed, SciELO, LILACS, EMBASE, and Web of Science, and in gray literature through Google Scholar. Studies were selected independently by two researchers using Rayyan software.

**Results:**

A total of 16 studies were included, of which 15 were scientific articles and one a doctoral thesis. Most of the CHE actions analyzed were carried out in person (56%), although technology-mediated online teaching and hybrid approaches have intensified in recent years. The studies covered a range of thematic areas, including family health, the prevention of sexually transmitted infections, child development, and the use of herbal medicines. The evaluation of the impact of CHE focused primarily on work process indicators – such as adherence to protocols, teamwork, and changes in professional practice – while health outcome indicators, such as improved prenatal care, vaccination coverage, and control of chronic diseases, were less frequently analyzed.

**Conclusion:**

Continuing health education has a positive impact on the training of primary care professionals and the qualification of health services. However, measuring the effects of continuing education on health outcomes remains incipient, pointing to the need for more robust studies that assess long-term impacts. In addition, the expansion of flexible strategies, such as technology-mediated teaching and hybrid approaches, can contribute to greater equity in access to professional training, aligning with the Sustainable Development Goals (SDGs) and strengthening primary care in Brazil.

**Pre-registration:**

The protocol for this review was registered with the Open Science Framework (OSF) under DOI [https://doi.org/10.17605/OSF.IO/784ED] – available at https://osf.io/784ed/.

## 1. Introduction

Continuing Health Education (CHE) in the Brazilian National Health System (SUS) requires significant efforts to promote and coordinate health services, educational institutions, and the work environment [1]. These efforts are particularly challenging due to the country’s territorial extension, cultural diversity, and regional asymmetries, as well as the large number of health workers—over four million—and approximately one hundred thousand health establishments opera`ting continuously [2]. Despite the progress made, challenges remain, including the need to further encourage the use of new educational technologies in the training of health professionals and to address the growing demands of public health.

In an attempt to respond to the constant need for training and updating professionals, in 2004, the Brazilian Ministry of Health proposed the National Policy for Continuing Education in Health (PNEPS) [3–5]. This initiative seeks to promote advances in health work, especially in the training of human resources, by restructuring educational practices in the field of health [6].

It is important to highlight that, in the Brazilian context, Permanent Health Education (Educação Permanente em Saúde) goes beyond the traditional concept of continuing education. It is a political-pedagogical approach that integrates education, healthcare services, and management, aiming to transform health practices based on the concrete needs of each territory [5].

The policy is based on activities that are part of everyday work and the realities experienced by professionals, using reflection on practice as a tool for qualifying and solving problems faced on a daily basis [7–9]. With the creation of the Policy, continuing education began to play a central role in the construction of knowledge and in the transformation of training and assistance processes [10].

Primary Health Care (PHC), recognized as the main gateway to the SUS, plays a strategic role in promoting health, preventing diseases, and managing comprehensive care, being capable of solving around 85% of the population’s health problems [11–13]. However, for this level of care to effectively achieve its objectives, it is essential that professionals are continually trained to deal with the challenges imposed by epidemiological, social, and technological changes [14].

In the context of PHC, continuing education stands out as an approach that incorporates the principles of problematization, contextualization of reality, innovative pedagogies, and reflective thinking [14]. In this way, it guarantees the updating of knowledge and the improvement of health workers’ skills, directly impacting the quality of the services provided [15]. In addition, by focusing on the problems that emerge from the daily lives of health teams, Continuing Health Education has promoted changes in the reality of the population in the territories [5,8,16,17].

Although Continuing Health Education (CHE) is essential for the qualification of health professionals, studies indicate that the evaluation and monitoring of CHE actions are still insufficient [18,19]. Furthermore, even with its great potential to impact the quality of care, there is still a gap in the literature regarding the real effects of CHE on improving care practice in PHC. In this context, there is a need to monitor and measure the effects of the actions implemented, as well as the impact of training processes on the provision of health services and on meeting the targets of the Sustainable Development Goals (SDGs) of the United Nations (UN) 2030 Agenda [20,21].

Thus, this study aims to identify and analyze evidence on the impact of Continuing Health Education for health professionals in PHC in Brazil. Specifically, this scoping review seeks to map the thematic areas addressed, the methodological strategies employed, and the indicators used to measure their impacts, contributing to the improvement of continuing education policies and practices in the country.

## 2. Materials and methods

This study was pre-registered in the Open Science Framework (OSF) under DOI [https://doi.org/10.17605/OSF.IO/784ED] (available at https://osf.io/784ed/).

This is a scoping review that aims to answer research questions, based on a rigorous, transparent and reliable synthesis of knowledge, guided by the methodology proposed by the Joanna Briggs Institute – JBI [22] and its reporting was guided by the Systematic Reviews and Meta-Analyses extension for Scoping Reviews (PRISMA-ScR) [23] to ensure transparency and methodological rigor.

The choice of this method is based on the need for a broad mapping of the literature on the subject. The methodological design of this study consists of six consecutive stages, as described in the previously published research protocol [24]: (1) define of the objectives and scope of the review; (2) identifying search terms; (3) establishing inclusion and exclusion criteria; (4) extracting data; (5) analyzing the results; and (6) presenting the findings.

It is important to note that this study did not directly involve human participants, and ethical approval was not required.

### 2.1. Step 1: Define of the objectives and scope of the review

Considering the objective of this study, it was structured according to the PCC mnemonic – (Population – health professionals; Concept – continuing education; Context – primary health care).

Thus, the general objective of this scoping review was to identify evidence on the impacts of CHE initiatives directed toward health professionals working in Primary Health Care. Specifically, the review aimed: (1) to map the thematic areas addressed in CHE initiatives for PHC professionals; (2) to describe the methodological strategies used in CHE activities within the PHC context; and (3) to identify the work process and health outcome indicators used to measure the impacts of these educational strategies.

### 2.2. Step 2 – Choosing search terms

The search strategies were designed according to each database, following keywords from three controlled health vocabularies (DeCS, MeSH, and Emtree), together with Boolean operators to obtain a broader result. Non-controlled words were also used to increase the sensitivity of the strategy. Articles identified in the references of the studies selected by the searches carried out and which are related to the objective of this review could be included.

The databases included in this study were MEDLINE (via PubMed), SciELO, LILACS, Embase, and Web of Science. The selection of these databases was justified by their high credibility, as they index journals of recognized academic reputation and are widely used as references for scientific research in the field. Google Scholar was also considered for the analysis of the first 100 documents retrieved, which were evaluated as potential sources of gray literature relevant to the objectives of this review. The literature searches were conducted in June 2024.

The complete search strategy, including all keywords, descriptors, and Boolean combinations used for each database, is available in Supplementary File 2.

The literature searches were conducted by one of the authors (LKMP)

### 2.3. Stage 3 – Definition of inclusion/exclusion criteria

The inclusion and exclusion criteria were structured according to the PCC framework: Population (P) – health professionals; Concept (C) – continuing education; and Context (C) – primary health care (PHC).

We chose to include publications from primary studies and gray literature: books, manuals, protocols, short communications, dissertations, and theses, which deal with continuing education for health professionals in PHC in Brazil, and which answer the question of the study. The survey included papers available in full and in electronic format, without language limitations, and published between 2007 and 2024. Duplicate publications, letters to the editor, expanded abstracts, editorials, and articles that were unavailable even after contacting the authors were excluded.

### 2.4. Step 4 – Data extraction

The process of selecting studies was guided by the path proposed in the Preferred Reporting Items for Systematic Review and Meta-Analyses (PRISMA-ScR) [23]. The Rayyan management software (Rayyan Systems Inc., Doha, Qatar) was used as a tool to support the selection of studies. Two independent reviewers (LKMP and EELCF) conducted the screening in two sequential stages: (1) title and abstract screening and (2) full-text review, both against the predefined inclusion/exclusion criteria.

The studies selected by title and abstract were retrieved in full and exported to a database in the Microsoft Excel® program. Discrepancies between the two reviewers at any stage were discussed, and when consensus could not be reached, a third reviewer (JAS) acted as arbiter. The paired review ensured the relevance of the studies and took place from July to September 2024.

After reading the full texts and validating the final sample, an assessment of the compatibility and relevance of the evidence with the aim of the review was performed.

**Fig 1:**
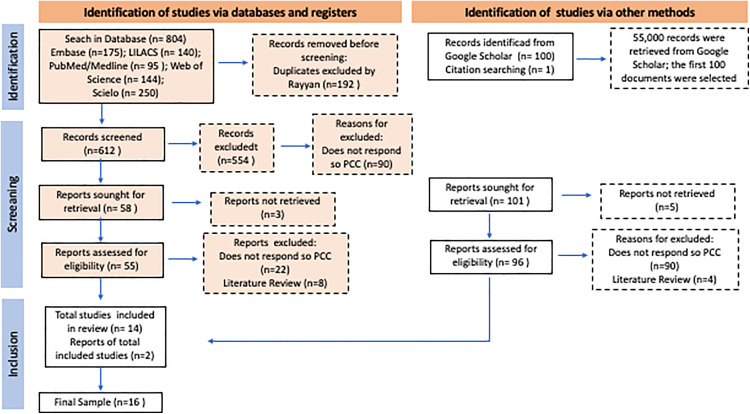
Selection of sources of evidence (adapted PRISMA 2020). Source: Research data, 2024.

A flowchart (Fig 1) was drawn up containing information on the screening and selection of studies included and excluded from this scoping review. Data extraction was performed independently by two reviewers (LKMP and EELCF) using a structured form developed by the authors, as presented in the published protocol [24]. The form included the following fields: publication title, authors, year of publication, country, language, study design, study population, study objectives, addressed themes, methodology used, and indicators of work process and health outcomes. Any discrepancies identified between the reviewers were discussed and resolved by consensus, with no need for third reviewer intervention.

It is worth noting that a pilot test was conducted before starting the study selection and data extraction to ensure alignment of the procedures. Both reviewers independently evaluated a random sample of 25 articles, assessing titles and abstracts according to the eligibility criteria. Afterwards, they met to discuss and resolve discrepancies and make the necessary adjustments to the criteria and definitions. Data extraction began only when 75% or more similarity was achieved between reviewers, ensuring methodological consistency and reliability.

### 2.5. Stage 5 – Analysis of the results

The mapped studies were analyzed descriptively, using the information described on the form used to extract the data and other characteristics considered relevant to the findings. The results of the studies were categorized into three analysis groups: a) topics covered; b) methodologies used; c) work process and/or health outcome indicators used to measure impact.

### 2.5. Stage 6 – Presentation of results

The results of the research are presented in a flowchart (Fig 1) and tables (Tables 1 and 2). The results were presented to experts in the field and analyzed in the light of the relevant literature in order to qualify the preliminary findings.

**Table 1:** Characterization of the studies included in the scoping review.

AUTHOR AND YEAR	TITLE	OBJECTIVE	TYPE OF STUDY/ APPROACH/ STUDY POPULATION	MAIN RESULTS OF THE STUDY
Santos et al. 2019 [25]	Impact of distance education on primary health care indicators in central Brazil: An ecological study with time trend analysis	The objective of this study was to verify whether the inclusion of professionals with a distance specialization course in family health teams is associated with hospitalization rates for primary care-sensitive conditions and better monitoring of chronic conditions in municipalities in the state of Mato Grosso do Sul, Brazil.	Ecological study/ Quantitative/ Primary Health Care Professionals	The indicators of the proportion of hospitalizations due to primary care-sensitive conditions (general rate and specific rates for asthma, gastroenteritis and heart failure) decreased during the study period when related to a high and intermediate proportion of professionals who completed the specialization course, and the same was observed for the indicators of chronic conditions (diabetics and hypertensives) who were registered, monitored and treated in a group.
Do Nascimento et al. 2020 [26]	Impact of continuing education on maternal and child health indicators	To investigate whether the presence of health professionals with a specialization course in family health was associated with improved care and maternal and child health indicators in municipalities in the state of Mato Grosso do Sul, Brazil.	Ecological study/ Quantitative/ Professionals who completed the specialization course in Family Health and provided care to the population in PHC	The results show that the training had a visible impact on the FSS process, with better indicators related to maternal and child health. Enrolment of pregnant women, exclusive breastfeeding for children under 4 months and up-to-date vaccinations for children under 1 year to 23 months increased in the municipalities where the professionals who completed the specialization course worked. There was also an increase in the intermediate ratio in the indicators related to cervical cancer screening and new diagnoses of congenital syphilis in children under one year old.
Fontoura 2017 [27]	Educational action, management and practice: implications for changes in the health care model	To analyze the relationship between the educational process and the work process of FHP doctors and nurses in the context of health system management in a municipality in the state of Bahia in 2006.	Case Study/ Qualitative/ Doctors and Nurses	They showed a positive relationship between the change in the profile of the professionals studied and the work-centered educational process they experienced.
Braun et al. 2021 [28]	Continuing education activities improve dentists’ self-efficacy to manage oral mucosal lesions and oral cancer	To assess whether Continuing Education Activities (CEA) influence dentists’ behavior in relation to oral lesions. The secondary objective was to assess the association between dentists’ perception of the adequacy of learning and self-efficacy for the management of oral mucosal lesions.	Cross-sectional study/ Quantitative/ Dental surgeons	Continuing education activities can improve primary health care professionals’ awareness and the effectiveness of oral cancer detection. Among dentists who frequently detected oral lesions, 88.9% had participated in CEA, while 11.1% had never participated in these activities.
Balogh et al. 2015 [29]	Care of adults with developmental disabilities: Effects of a continuing education course for primary care providers	To evaluate the effects of an interdisciplinary continuing education course based on guidelines on measures related to the care of adults with developmental disabilities (DD).	Intervention study/ Quantitative/ Doctors, dentists, and nurses	The results indicated that, compared to a group of primary care providers who did not take the course, providers who took the continuing education course had better results in 4 out of 5 key measures: frequency of use of the Guidelines and Tools Book, frequency of conducting assessments when a patient exhibits a change in behavior, comfort level when caring for adults with DD and knowledge of primary care related to adults with DD.**
Haraguchi et al. 2020 [30]	Impact of the Training of Professionals from São Paulo Public Health System in Phytotherapy Practice	To evaluate the impact of the 2014 and 2015 editions of the “Medicinal Plants and Herbal Medicines” course on the practices of health professionals.	Exploratory and descriptive study/ Quali-quantitative/ Biomedical, Dental surgeon, Nurse, Pharmacist, Physiotherapist, Doctor, and Nutritionist	The course had a positive impact on the acceptance and application of phytotherapy by health professionals, with a significant increase (p < 0.001) in the expansion of activities related to phytotherapy (“tea” circles, “medicinal gardens” and training). There was also an impact on the use of herbal products such as Matricaria chamomilla, Maytenus ilicifolia and Valeriana officinalis. There was also an increase in awareness of the risks of herbal medicine, although without a corresponding increase in the reporting of adverse reactions.
Lazarini e Barbosa 2017 [31]	Educational intervention in Primary Care for the prevention of congenital syphilis	To assess the effectiveness of the educational intervention on the knowledge of primary care health professionals and to verify the impact on vertical transmission rates of congenital syphilis.	Quasi-experimental study/ Quantitative/ Doctors, Nurses, and Nursing Technicians	The average number of correct answers rose from 53% to 74.3% after the intervention (p < 0.01). Adherence to professional training was 92.6%. There was a significant reduction in the rate of vertical transmission of syphilis from 75% in 2013 to 40.2% in 2015. In 2014 and 2015, there were no records of infant mortality due to this condition.
Mattos; Dahmer e Magalhães 2015 [32]	Contribution of the specialization course in primary health care to the practice of health professionals	To analyze the contribution of a specialization course in Family Health, in both face-to-face and distance learning modalities, to the practice of health professionals in the state of Rio Grande do Sul.	Intervention study/Qualitative/ Doctors, Dentists, and Nurses	The course brought about changes, such as reorganizing the team’s work process, implementing collective activities, and welcoming spontaneous demand, as well as a greater understanding of the work process.
Amaral et al. 2014 [33]	Impact of training health professionals on cervical cancer screening in basic health units	To evaluate the impact of training professionals involved in cervical cancer (CC) screening in Basic Health Units (UBSs) in the municipality of Goiânia (GO).	Intervention study/ Quantitative/ Doctors, Nurses, Nursing Technicians, and Community Health Workers.	After the training, there was a significant improvement in filling in the requisition form, in carrying out the cytopathology test according to the periodicity and age range recommended by the Ministry of Health, and in the adequacy of the sample.
Sousa et al. 2023 [34]	Continuing education on child development in primary care: healthcare workers’ perspectives.	To analyze the contributions of continuing education with primary health care professionals who promote child development.	Intervention study/ Qualitative/ Health professionals responsible for the care of children aged 0–6 in a Primary Care service	The results of the study highlight the significant impact of such interventions on changing professional perceptions and practices related to child development. Overall, this research provides valuable information on the effectiveness of continuing education interventions to promote healthy child development in primary care settings
Cardoso et al. 2022 [35]	Distance learning course improves primary care dentists’ diagnosis and self-efficacy in the management of oral lesions	To evaluate the impact of a distance learning course on the diagnosis of oral mucosal lesions offered to public health dentists.	Quasi-experimental study/ Quantitative/ Dental surgeons	The classification of the nature of the lesions, diagnostic hypotheses, sensitivity and specificity improved by 13.4%, 10.0%, 13.4% and 6.6% respectively (p < 0.01, Wilcoxon test). With regard to management, there was a 16.6% reduction in the intention to refer cases, while confidence in the diagnosis of benign lesions increased by 40%.
De Mendonça et al. 2017 [36]	Evaluation of a training course: implications for practice	Identify the effects of an educational action at work after 120 days	Action-research study/ Qualitative/ Primary Health Care professionals	The opinion of the participants regarding the effects of the CHE on their work showed that the activity promoted changes in the actions carried out. After 120 days of training, the professionals reported a re-reading of the groups held in PHC, with a diversification of the resources used, and recognized that after the educational activity, they were able to manage the groups with more knowledge, safety, and respect for the elderly.
Figueiras; Puccini e Silva 2014 [37]	Continuing education on child development for primary healthcare professionals: a prospective before-and-after study	To evaluate the impact of a continuing education program on child development and on the knowledge and practices of these professionals.	Prospective single cohort study (before and after study)/ Quantitative/ Primary Health Care professionals	From one to three years after the program, the average number of correct answers increased from 11.5 to 14.3 among doctors from the Healthy Family Program (PFS); from 13.0 to 14.3 among doctors from Municipal Health Units (UMS); from 8.3 to 10.0 among PFS nurses; and from 7.8 to 9.4 among UMS nurses. In interviews with mothers seen by these professionals before the program, only 21.7% reported that they were asked about their children’s development, 24.7% reported that the professional asked or observed their children’s development, and 11.1% received guidance on stimulating them. After the program, these percentages increased to 34.5%, 54.2%, and 30.3%, respectively
Da Silva Cais et al. 2011 [38]	Suicide Prevention Training for Professionals in the Public Health Network in a Large Brazilian City	Measuring the impact of training in terms of change in the knowledge and attitudes of health in relation to suicidal behavior.	Quantitative/ Primary Health Care professionals who were routinely involved with patients at high risk of suicide.	The score on the knowledge questionnaire, with 21 points as the maximum value, increased from 8.9 to 13 points (p < 0.001, 95% significance level). Of the 25 questionnaire items representing attitudes, 18 showed a significant change after the training. This training model increased knowledge and attitudes towards suicide prevention in healthcare professionals.
Ricardo et al. 2022 [39]	Virtual Learning Environment of the Brazilian Health System (AVASUS): Efficiency of Results, Impacts, and Contributions	Evaluate the impact of educational offers on health services and the professional practice of AVASUS course participants.	Quantitative/ Health professionals	The results showed that 76.2% of respondents recommended AVASUS courses to their peers. Thus, the quality of the educational offerings motivated 81.3% of these recommendations. In addition, 75.6% of the course participants who answered the questionnaire also indicated that the contents of the AVASUS course contribute to improving the existing health services in the health units where they work. Finally, 24.6% of all responses mentioned that the courses available on AVASUS were essential for offering new health services in these units.
Caitano et al. 2022 [40]	Massive health education through technological mediation: Analyses and impacts on the syphilis epidemic in Brazil	To analyze the impacts of the “Syphilis and other STIs” learning pathway on the response to the epidemic in Brazil, highlighting the educational process of the learning pathway and its social implications from the perspective of the United Nations 2030 Agenda and its Sustainable Development Goals.	Quantitative/ Health professionals	It was found that, as the number of children enrolled increased from 2018 onwards, vertical transmission of syphilis began to fall. This positive impact on vertical transmission rates indicates resilience and responsiveness due to changes in SUS work processes.

Source: Research data, 2024.

**Table 2:** Thematic areas, methodological strategies, and indicators of work process and health outcomes identified in the included studies.

AUTHOR AND YEAR	THEMATIC AREA	METHODOLOGICAL STRATEGY	WORK PROCESS INDICATORS	HEALTH OUTCOME INDICATORS
Santos et al. 2019 [25]	Family Healthcare	Distance learning specialization course.		Hospitalization rates for primary care-sensitive conditions; Rates of diabetic patients registered and monitored in primary care; Rates of hypertensive patients registered and monitored in primary care.
Do Nascimento et al. 2020 [26]	Family Healthcare	Distance learning – Workload 405 hours		Number of pregnant women; Number of prenatal consultations; Number of children exclusively breastfed up to 4 months of age; Number of children under 1 year old and children aged 12–23 months with up-to-date vaccinations; Number of preventive examinations for cervical cancer; Number of maternal deaths during a given period and place of residence; Number of cases of congenital syphilis in children under one year old.
Fontoura 2017 [27]	Family Healthcare	Specialization course in Family Healthcare – total workload of 690 hours distributed in one (1) introductory module and six (6) sequential face-to-face modules, of which there were 330 hours of theory and 360 of practice, computed in the work process itself.	Individual assistance; Home visits; Group activities, with specific groups and educational activities in the waiting room; Team and unit meetings.	
Braun et al. 2021 [28]	Oral mucosal lesions	“Red May Project” - face-to-face meetings, with oral presentations and delivery of educational materials	Frequency of oral mucosa examination; Frequency of lesions detected; Frequency of oral cancer cases detected; Number of continuing education courses attended on oral cancer.	Number of diagnoses of oral lesions; Number of biopsies of oral lesions; Number of treatments for oral lesions.
Balogh et al. 2015 [29]	Caring for adults with disabilities	Training process structured around case scenarios and consisted of 3 online modules; there were also 2 full-day face-to-face workshops (1 workshop at the beginning and 1 at the end of the course).	Frequency of use of guidelines; Frequency of regular health examinations; Frequency of evaluating patients who exhibit changes in behavior, comfort level when caring for adults with Developmental Disabilities (DD); Knowledge of primary care related to adults with DD.	
Haraguchi et al. 2020 [30]	Medicinal plants and herbal medicines	Face-to-face meeting – 6 months, every Friday	• Acceptance and application of phytotherapy by health professionals.	
Lazarini e Barbosa 2017 [31]	Syphilis in PHC	Workshops – held in 3 stages	Diagnosis of gestational and congenital syphilis Management of gestational and congenital syphilis Collective activities Reception of spontaneous demand Reorganizing the work process (team meetings and organizing agendas)	Rate of vertical transmission of syphilis Infant mortality rate due to this disease.
Mattos; Dahmer e Magalhães 2015 [32]	Family Healthcare	On-site and distance learning specialization course – active methodology	Diagnosis of gestational and congenital syphilis Management of gestational and congenital syphilis Collective activities Reception of spontaneous demand Reorganizing the work process (team meetings and organizing agendas)	
Amaral et al. 2014 [33]	CC screening in PHC	Face-to-face meeting	Filling in the request form. Carrying out the cytopathological examination, Suitability of the sample.	
Sousa et al. 2023 [34]	Child development in PHC	Eight face-to-face workshops – active methodology	Acquisition of knowledge about child development; Perception of improvement in professional practices related to child development	
Cardoso et al. 2022 [35]	Diagnosis and management of oral lesions in PHC	Distance learning – 50 hours spread over 10 units over 3 months. Problem-based learning (PBL)	Specificity and sensitivity in the diagnosis of oral mucosal lesions; Referral of cases of oral lesions.	
De Mendonça et al. 2017 [36]	Health education for the elderly.	Four weekly meetings lasting four hours each – active methodology.	• Management in the elderly group	
Figueiras; Puccini e Silva 2014 [37]	Child development	20h classroom course – includes lectures and discussion of the topic	Carrying out routine assessments of the development of children attending the service; Providing guidance to mothers on how to stimulate their children’s development.	
Da Silva Cais et al. 2011 [38]	Suicide prevention	Face-to-face training – theoretical exposition and discussion of clinical cases	Management of depressed patients; Ability to assess suicide risk; Epidemiological information on suicidal behavior; Diagnosis and management of alcohol-dependent individuals; Knowledge of effective suicide prevention strategies; Ability to assess lethal intent in attempted suicide; Knowledge of the genesis of suicidal behavior and the acute mental functioning of individuals at risk of suicide.	
Ricardo et al. 2022 [39]	Educational offers registered with AVASUS	Distance learning	Acquisition and application of knowledge obtained through the courses/modules Course recommendations from the courses/modules	
Caitano et al. 2022 [40]	Syphilis and other STIs	Distance learning		Number of notifications of syphilis cases. Syphilis testing data.

Source: Research data, 2024.

## 3. Results

The initial database search yielded 804 records while an additional 101 records were identified through other methods, including 100 from the gray literature and 1 through citation searching.

A total of 192 duplicate records were removed from the study. Subsequently, a screening process was carried out, considering all records retrieved from both databases and other sources. During this stage, eight studies were excluded because their full texts were not available. Among the remaining exclusions, 112 publications did not meet the PCC criteria, and 12 were literature reviews. It should be noted that one article [25], related to the aim of this review, was included from the references of a study [26] selected in the searches. In total, 16 documents were included in the final sample, comprising 15 scientific articles and 1 doctoral thesis [27]. The search results and the study selection process are summarized in Fig 1.

The characterization of the studies included in the scoping review, according to author, year, title, objective, type of study, approach, study population, as well as the main results of the studies are shown in Table 1.

The mapping of the sources that make up the results of this study showed that of the total of 16 documents, 56% (9) were publications from the period 2007–2019 [25, 27, 29, 31 - 33, 36 - 38] and 44% (7) were publications from the last 5 years (2020–2024) [26, 28, 30, 34, 35, 39, 40].

Although the highest percentage of evidence was obtained before the last five years, interest in studying the impact of health professionals’ CHE actions in PHC has increased, especially in recent years.

We identified three intervention-type studies [29, 32, 33, 34], three analytical studies [38–40], one case study [27], one cross-sectional study [28], one exploratory-descriptive study [30], two quasi-experimental studies [31,35], two ecological studies [25, 26], one prospective study [37] and one action research study [36]. As for the methodological approach used, eleven studies were quantitative [25, 26, 28, 29, 31, 33, 35, 37 - 40], four were qualitative [27, 32, 34, 36], and one was mixed [30].

With regard to the population of the studies, it was possible to identify in the findings the participation of various categories of health professionals working in PHC [25,26,34,36-40], such as Biomedical, Dental Surgeon, Nurse, Pharmacist, Physiotherapist, Physician, and Nutritionist. Other studies were aimed more at university-educated professionals who make up the minimum team of the Family Health Strategy (eSF), namely doctors, nurses, and dentists [27-30,35]. The studies by Lazarini and Barbosa [31] and Amaral et al [33] also included technical (nursing technicians) and medium-level (community health workers) professionals.

With regard to the CHE actions described in the selected studies, the mapping of published information was categorized according to the thematic areas covered, the methodological strategies used, and the work process and health outcome indicators used to measure the impact of the training strategy, as described in Table 2.

Based on the publications analyzed, the thematic areas covered in continuing education actions for health professionals in PHC include: family health [25 - 27, 32], oral mucosa lesions [28, 35], care for adults with disabilities [29], medicinal plants and herbal medicines [30], syphilis and/or other STIs [31, 40], cervical cancer [33], child development [34, 37], health education for the elderly [36], and suicide prevention [38]. The study by Ricardo et al [39] covers various subject areas in courses offered in a virtual learning environment.

Among the studies selected in this review, the majority (n = 9) deal with educational actions implemented through face-to-face modality [27, 28, 30, 31, 33, 34, 36-38], using methodological strategies such as: oral presentations, workshops, and presentation of clinical cases. In addition, distance learning actions were identified in six studies [25, 26, 35, 39, 40]. The study by Balogt and colleagues [29] presented an intervention with a hybrid approach, combining a technology-mediated course and two face-to-face workshops as methodological strategies. Mattos, Dahmer, and Magalhães [32] carried out a study based on a face-to-face educational process and a technology-mediated one.

To measure the impact of CHE actions, the studies analyzed adopted different measures. Eleven studies used exclusively work process indicators [27, 29 30,32 - 39], while three studies focused on health outcome indicators [25, 26, 40]. Two studies used work process and health outcome indicators, seeking a more comprehensive assessment of the impact of Continuing Education on health services [28, 31]. Among the analyzed studies, process indicators were predominant, reflecting changes in professional practice and organization of work. In contrast, health outcome indicators, such as those related to maternal and child health or disease prevention, were less commonly evaluated.

## 4. Discussion

This review presents us with a comprehensive mapping of CHE actions aimed at PHC professionals in Brazil. The study highlights the various thematic areas, methodological strategies and impact indicators used, which provides a broad view of how CHE is being implemented and evaluated in the context of PHC. In addition, the review identifies emerging trends, such as the growing use of technology-mediated teaching, and highlights gaps and opportunities for future research, especially in relation to direct impacts on the population’s health outcomes.

The analysis revealed that Continuing Education in Health has an impact on work processes in Primary Health Care, mainly in terms of qualification and updating knowledge, demonstrated by the predominance of studies that evaluated the effects and impacts on work processes and found positive results [27, 29, 30, 32–39]. It was observed that these processes were evaluated on the basis of changes in professional practice following continuing education.

Most of these studies followed this approach, and carried out measurements before and after educational actions to identify changes in performance or professional practices, such as the study by Cardoso et al [35], which applied a pre-test and post-test for dental surgeons after a 3-month online course on the diagnosis and management of oral lesions in Primary Health Care. The study showed that the classification of the nature of the lesions, diagnostic hypotheses, sensitivity, and specificity improved by 13.4%, 10.0%, 13.4%, and 6.6%, respectively [35].

The perception of professionals was also used as a parameter to measure the impact of continuing education processes. In the research carried out by Mattos; Dahmer and Magalhães [32], according to the perception of doctors, dentists and nurses, after the training process focused on Family Health, there was a reorganization of the team’s work process, implementation of collective activities and reception of spontaneous demand, as well as a greater understanding of the work process.

It is important to note that the literature emphasizes how health professionals are not always trained to approach the health-disease process with a comprehensive and integrated perspective. This limitation often results in fragmented and low-resolution practices, which can reduce the effectiveness of outcomes in Primary Health Care [41].

From the studies reviewed, it was observed that the measurement of health outcomes as an evaluation of Continuing Health Education processes is still incipient, and is an area that needs greater attention in future research [42,43]. This contrasts with the predominance of work process indicators found in most studies, which assess immediate changes in professional practice and service organization. Such distinction helps to contextualize how CHE has been measured in Brazil, emphasizing the gap in evaluating long-term health results.

According to the literature, the evaluation processes of Continuing Education in Health are still poorly implemented in Brazil [44]. In general, these initiatives are isolated and occasional, although they play an important role in health policy by enabling the measurement of an intervention’s degree of implementation, its effects, and impacts [45]. In this sense, monitoring and evaluating continuing education actions is both a major shortcoming and a major necessity for strengthening the National Policy for Continuing Education in Health and, consequently, for consolidating the SUS [46].

The use of health indicators is one of the main evaluation measures in public health, qualifying services and allowing for the analysis of the population’s life process. It is essential, among other factors, for demonstrating the impact of interventions on the population’s health, as well as evaluating subjective concepts such as care and access [47]. In this context, the incorporation of results indicators in the evaluation of CHE practices not only makes it possible to measure the improvement of work processes but also provides robust data for decision-making and the allocation of resources in health management.

The relationship between CHE and the monitoring of health indicators is an indispensable complementary strategy for boosting the impact of actions in PHC, as we can see in the studies that measured impact based on health outcomes. Nascimento and collaborators [26] investigated whether taking a specialization course in Family Health, in the online modality, was associated with an improvement in care and maternal and child health indicators. They used parameters such as the monitoring of pregnant women, children breastfed for up to 4 months, preventive examinations for cancer and cervical cancer, as well as the diagnosis of new cases of the disease. The results show that the training had a visible impact on the CHE process, with better indicators related to maternal and child health [26].

The study by Santos and collaborators [25], In 2019, analyzed the impact of a specialization course in Family Health, focusing on the insertion of qualified professionals and its relationship with hospitalization rates for primary care-sensitive conditions, in addition to the monitoring of chronic conditions in a municipality. The results indicated a reduction in the overall and specific rates of hospitalizations for primary care-sensitive conditions (including asthma, gastroenteritis, and heart failure) over the period studied, especially in areas with a high or intermediate proportion of professionals who had completed specialization. Similarly, there was an improvement in monitoring indicators for chronic conditions such as diabetes and hypertension, with greater registration, follow-up, and group care for these patients [25].

The study by Caitano et al [40], published In 2022, analyzed the impact of a training course on syphilis and other sexually transmitted infections. The study focused on evaluating the results of this training process in the management and prevention of these diseases based on epidemiological data and the number of tests. It was found that, as the number of children enrolled increased from 2018 onwards, vertical transmission of syphilis began to fall. This positive impact on vertical transmission rates indicates resilience and responsiveness due to changes in the work processes of the Brazilian National Health System (SUS) [14].

The thematic areas covered by the Continuing Health Education (CHE) actions are diverse, ranging from general aspects of Family Health to more specific topics, such as oral mucosal lesions, care for people with disabilities, suicide prevention, and health education for the elderly. This variety of topics highlights the flexibility of continuing education, which adjusts to local needs and the demand for specialized training within Primary Health Care, considered a dynamic and complex health scenario [48]. According to Ferreira (2019) [49], CHE has the work process as the object of transformation in the practice scenario, based on the professionals’ critical reflection on what is happening in the daily routine of the services and seeking solutions to the problems encountered together with the team.

The studies analyzed showed that the methodological strategies used are predominantly face-to-face. However, the use of technology-mediated teaching has intensified in recent years, which reflects the need for more flexible strategies that show promise for expanding the reach and effectiveness of training processes [50, 51]. The study by Ricardo Valentim and collaborators (2022) [39] evaluated the impact of online educational offers on health services and the professional practice of course participants. The results indicated that 76.2% of the study participants recommended the courses, and 81.3% of these recommendations were motivated by the quality of the course. In addition, 75.6% of respondents indicated that the content of the courses contributed to improving health services in the health establishments where they work.

The adoption of hybrid approaches, combining face-to-face teaching with technology-mediated teaching, also proved to be a viable alternative. It was observed that face-to-face activities, with workshops preceded by online activities in virtual environments, was the main association adopted in a training process, which had a positive impact, promoting changes in professionals’ perceptions and practices about child development in primary care settings [29].

The growing use of technology-mediated education and digital tools in Continuing Health Education is in line with the evolution of digital health [52], which seeks to integrate technologies to improve the quality and efficiency of health services. The use of digital platforms allows health professionals to access educational content more flexibly, overcoming geographical and time barriers [51, 53].

Regarding health professionals, the findings of this review also show that Continuing Health Education in PHC has a significant reach, as it involves professionals from different categories [54, 55]. In addition, the inclusion of nursing technicians and Community Health Agents (CHAs) in educational actions highlights an advance in valuing these professionals, who play a crucial role in promoting health and monitoring the population, particularly in Brazil, where there is an appreciation of the participation of these two professionals in PHC.

This diversity reflects the need for interdisciplinary training that favours integration between professionals and improves teamwork, an essential factor in guaranteeing quality care in PHC [56, 57]. Continuing Health Education strategies that promote collaboration between different areas of knowledge increase the problem-solving capacity of services and strengthen user-centred care [58].

Mendes [12] already confirmed these findings in 2012 by demonstrating the positive impact of multiprofessional teamwork on various dimensions of PHC practice: on user experiences; on the professional development of health team members; on the quality of care and health outcomes; on the utilization of health resources; and on provider satisfaction. In this context, considering that professionals do not arrive fully prepared from their training institutions and require continuous in-service education, it becomes essential to implement a culture of collaborative and humanized practice in PHC.

When reflecting on the findings of this review, it is evident that Continuing Health Education appears to contribute to transforming work processes and may favor improvements in health outcomes, in alignment with the principles promoted by the Sustainable Development Goals (SDGs) — particularly SDG 3 (Good Health and Well-being), SDG 4 (Quality Education), SDG 8 (Decent Work and Economic Growth), and SDG 10 (Reduced Inequalities) [59,60].

The findings reinforce the relevance of expanding training opportunities, especially through technological mediation and hybrid approaches, which seem to facilitate broader access to education for professionals in different regions and contexts [59–61]. Such strategies support the strengthening of Primary Health Care and the promotion of equity in the professional qualification process, contributing indirectly to the advancement of the 2030 Agenda.

Therefore, investing in Continuing Health Education may be considered a structuring component in the qualification of the health workforce, contributing to improved performance of health services and to the sustainability of health systems, particularly within the Brazilian context.

Although the findings of this review provide relevant insights into Continuing Education in PHC, some limitations should be considered. The selection of studies was restricted to the selected sources, which may have excluded relevant publications outside these sources. In addition, most of the studies analyzed focused on work process indicators, without a more in-depth assessment of health outcomes, which limits the understanding of the real impact of Continuing Education in Health in primary health care.

This review advances current knowledge by providing an updated and comparative synthesis of Brazilian evidence on the impacts of Continuing Health Education. It highlights persistent gaps in measuring health outcomes and in the methodological approaches used in national studies.

## 5. Implications for practice, policy, and future research

His scoping review offers relevant contributions by synthesizing national evidence on the impacts of Continuing Health Education (CHE) in Primary Health Care (PHC) in Brazil. The findings demonstrate that CHE initiatives predominantly influence work process indicators, such as changes in professional practices, organization of services, and adherence to protocols, while the evaluation of health outcome indicators remains limited.

From a practical and managerial perspective, these results highlight the importance of structuring continuing education programs that are closely aligned with daily work processes in PHC, fostering reflective practice and team-based organization. For policymakers, the findings reinforce the need to strengthen evaluation strategies within the National Policy for Continuing Health Education, incorporating health outcome indicators to better assess the effectiveness and sustainability of educational interventions.

Regarding future research, this review underscores the need for studies that adopt more robust and longitudinal designs, capable of linking continuing education initiatives to measurable health outcomes. Advancing this agenda may contribute to more evidence-informed decision-making and to the development of more effective continuing education strategies in Primary Health Care.

## 6. Conclusion

Continuing Health Education in Primary Health Care in Brazil has addressed several thematic areas, and its effects have been observed especially in changes in work processes. The findings of this scoping review indicate that most studies focus on process indicators, such as adherence to protocols and changes in professional practices, revealing a persistent gap in the assessment of impacts on health outcomes. Only a limited number of studies evaluated clinical outcomes, such as reductions in syphilis reporting or hospitalizations for primary care-sensitive conditions

Another relevant aspect identified was the predominance of in-person methodological strategies, although the growing adoption of technology-mediated and hybrid approaches in recent years signals an important transformation in continuing education practices. This movement reflects broader advances in digital health and highlights new possibilities for expanding access to professional training.

Overall, this review reinforces the relevance of Continuing Health Education as a strategic component for strengthening Primary Health Care in Brazil, while also emphasizing the need for more robust and comprehensive evaluation approaches. Future studies should prioritize the integration of process and outcome indicators in order to better capture the long-term effects of continuing education initiatives on health systems and population health.

## Supporting information

S1 Appendix PRISMA. (PDF)

S2 File Full Search Strategy for Databases and Gray Literature. (PDF)
