# Supplementary material for: Impacts of continuing education for health professionals in primary health care: A scoping review
Source: PLoS One. 2025 Dec 31;20(12):e0339980. doi: 10.1371/journal.pone.0339980 (PMC12755813; doi:10.1371/journal.pone.0339980)
Supplement: S1 Appendix — (PDF) [file pone.0339980.s001.pdf]

Preferred Reporting Items for Systematic reviews and Meta-Analyses extension for Scoping Reviews (PRISMA-ScR) Checklist

SECTION	ITEM	PRISMA-ScR CHECKLIST ITEM	REPORTED ON PAGE #
**TITLE**			
Title	1	Identify the report as a scoping review.	**Yes. The title is " impacts of continuing education for Primary Health Care professionals - a scoping review”**
**ABSTRACT**			
Structured summary	2	Provide a structured summary that includes (as applicable): background, objectives, eligibility criteria, sources of evidence, charting methods, results, and conclusions that relate to the review questions and objectives.	**Yes. The abstract is on page 2 of the manuscript.**
**INTRODUCTION**			
Rationale	3	Describe the rationale for the review in the context of what is already known. Explain why the review questions/objectives lend themselves to a scoping review approach.	**Yes. The review questions/objectives are on pages 5 and 6 of the manuscript.**
Objectives	4	Provide an explicit statement of the questions and objectives being addressed with reference to their key elements (e.g., population or participants, concepts, and context) or other relevant key elements used to conceptualize the review questions and/or objectives.	**Yes. The key elements (e.g., population or participants, concepts, and context) are on pages 5 and 6 of the manuscript.**
**METHODS**			
Protocol and registration	5	Indicate whether a review protocol exists; state if and where it can be accessed (e.g., a Web address); and if available, provide registration information, including the registration number.	**Yes. The protocol was registered in the Open Science Framework (DOI 10.17605/OSF.IO/784ED).**
Eligibility criteria	6	Specify characteristics of the sources of evidence used as eligibility criteria (e.g., years considered, language, and publication status), and provide a rationale.	**Yes. The eligibility criteria is on page 07 of the manuscript, and more details in the published protocol - doi:10.1371/journal.pone.0312963**
Information sources*	7	Describe all information sources in the search (e.g., databases with dates of coverage and contact with	**Yes, this information can be found on pages 07 and 08 of the manuscript, and more details in the published**

SECTION	ITEM	PRISMA-ScR CHECKLIST ITEM	REPORTED ON PAGE #
		authors to identify additional sources), as well as the date the most recent search was executed.	**protocol - doi:10.1371/journal.pone.0312963**
Search	8	Present the full electronic search strategy for at least 1 database, including any limits used, such that it could be repeated.	**Yes. The complete electronic search strategy is provided on page 06, and in more detail in the published protocol - doi:10.1371/journal.pone.0312963**
Selection of sources of evidence†	9	State the process for selecting sources of evidence (i.e., screening and eligibility) included in the scoping review.	**Yes. The process for selecting sources of evidence is on page 07, and in more detail in the published protocol - doi:10.1371/journal.pone.0312963**
Data charting process‡	10	Describe the methods of charting data from the included sources of evidence (e.g., calibrated forms or forms that have been tested by the team before their use, and whether data charting was done independently or in duplicate) and any processes for obtaining and confirming data from investigators.	**Yes. The methods of charting data are on page 07, and in more detail in the published protocol - doi:10.1371/journal.pone.0312963**
Data items	11	List and define all variables for which data were sought and any assumptions and simplifications made.	**Yes. The list and definition are in Appendix 1 of the published protocol - doi:10.1371/journal.pone.0312963**
Critical appraisal of individual sources of evidence§	12	If done, provide a rationale for conducting a critical appraisal of included sources of evidence; describe the methods used and how this information was used in any data synthesis (if appropriate).	**No critical appraisal of individual sources of evidence will not be done.**
Synthesis of results	13	Describe the methods of handling and summarizing the data that were charted.	**Yes. The methods of handling and summarizing the data that were charted are on page 08, and in more detail in the published protocol - doi:10.1371/journal.pone.0312963**
**RESULTS**			
Selection of sources of evidence	14	Give numbers of sources of evidence screened, assessed for eligibility, and included in the review, with reasons for exclusions at each stage, ideally using a flow diagram.	**Yes. This information is presented on pages 08 and 09 of the manuscript.**
Characteristics of sources of evidence	15	For each source of evidence, present characteristics for which data were charted and provide the citations.	**Yes. This information is presented in Box 1 (page 09).**

SECTION	ITEM	PRISMA-ScR CHECKLIST ITEM	REPORTED ON PAGE #
Critical appraisal within sources of evidence	16	If done, present data on critical appraisal of included sources of evidence (see item 12).	**No critical appraisal of individual sources of evidence will not be done.**
Results of individual sources of evidence	17	For each included source of evidence, present the relevant data that were charted that relate to the review questions and objectives.	**Yes. This information is presented in Box 2 (page 13).**
Synthesis of results	18	Summarize and/or present the charting results as they relate to the review questions and objectives.	**Yes. This information is presented in the Results section, more precisely, from page 13 onwards.**
**DISCUSSION**			
Summary of evidence	19	Summarize the main results (including an overview of concepts, themes, and types of evidence available), link to the review questions and objectives, and consider the relevance to key groups.	**Yes. This information is presented in the Discussion section, more precisely, on pages 16 to 22.**
Limitations	20	Discuss the limitations of the scoping review process.	**Yes. This information is presented at the end of the Discussion section, more precisely, on page 23.**
Conclusions	21	Provide a general interpretation of the results with respect to the review questions and objectives, as well as potential implications and/or next steps.	**Yes. This information is presented in the Conclusions section, starting on page 23.**
**FUNDING**			
Funding	22	Describe sources of funding for the included sources of evidence, as well as sources of funding for the scoping review. Describe the role of the funders of the scoping review.	**The financial disclosure is " Ministry of Health - Brazil. Project: 085/2021."**

JBI = Joanna Briggs Institute; PRISMA-ScR = Preferred Reporting Items for Systematic reviews and Meta-Analyses extension for Scoping Reviews.

* Where *sources of evidence* (see second footnote) are compiled from, such as bibliographic databases, social media platforms, and Web sites.

† A more inclusive/heterogeneous term used to account for the different types of evidence or data sources (e.g., quantitative and/or qualitative research, expert opinion, and policy documents) that may be eligible in a scoping review as opposed to only studies. This is not to be confused with *information sources* (see first footnote).

‡ The frameworks by Arksey and O'Malley (6) and Levac and colleagues (7) and the JBI guidance (4, 5) refer to the process of data extraction in a scoping review as data charting.

§ The process of systematically examining research evidence to assess its validity, results, and relevance before using it to inform a decision. This term is used for items 12 and 19 instead of "risk of bias" (which is more applicable to systematic reviews of interventions) to include and acknowledge the various sources of evidence that may be used in a scoping review (e.g., quantitative and/or qualitative research, expert opinion, and policy document).

From: Tricco AC, Lillie E, Zarin W, O'Brien KK, Colquhoun H, Levac D, et al. PRISMA Extension for Scoping Reviews (PRISMAScR): Checklist and Explanation. Ann Intern Med. 2018;169:467–473. doi: 10.7326/M18-0850.
