# Supplementary material for: Impacts of continuing education for health professionals in primary health care: A scoping review
Source: PLoS One. 2025 Dec 31;20(12):e0339980. doi: 10.1371/journal.pone.0339980 (PMC12755813; doi:10.1371/journal.pone.0339980)
Supplement: S2 File — (PDF) [file pone.0339980.s002.pdf]

FULL SEARCH STRATEGY FOR DATABASES AND GRAY LITERATURE.

**Medline/PUBMED**	("permanent education"[Title/Abstract] OR "Continuous Learning"[Title/Abstract] OR "Lifelong Learning"[Title/Abstract] OR "life long learning"[Title/Abstract] OR "life long learning"[Title/Abstract] OR "life long learning"[Title/Abstract] OR "Continuing Education"[Title/Abstract]) AND ("Health Personnel"[Title/Abstract] OR "Health Care Providers"[Title/Abstract] OR "Health Care Provider"[Title/Abstract] OR "Health Care Provider"[Title/Abstract] OR "Healthcare Providers"[Title/Abstract] OR "Healthcare Provider"[Title/Abstract] OR "Healthcare Provider"[Title/Abstract] OR "Healthcare Workers"[Title/Abstract] OR "Healthcare Worker"[Title/Abstract] OR "Health Care Professionals"[Title/Abstract] OR "Health Care Professional"[Title/Abstract] OR "Health Care Professional"[Title/Abstract]) AND ("Primary Health Care"[Title/Abstract] OR "Primary Healthcare"[Title/Abstract] OR "Primary Care"[Title/Abstract])
**SCIELO**	((permanent education) OR (education continuing)) AND ((health professionals) OR (health care professionals) OR (health personnel)) AND ((primary health care) OR (primary healthcare) OR (primary care))
**WEB OF SCIENCE**	(“permanent education" OR “Continuous Learning” OR “Lifelong Learning” OR “Life-Long Learning” OR “Life-Long Learning” OR “Life-Long Learnings” OR “Life Long Learning” OR “Life-Long Learnings” OR “Continuing Education”) AND (“Health Personnel” OR “Health Care Providers” OR “Health Care Provider” OR “Health Care Provider” OR “Healthcare Providers” OR “Healthcare Provider” OR “Healthcare Provider” OR “Healthcare Workers” OR “Healthcare Worker” OR “Health Care Professionals” OR “Health Care Professional” OR “Health Care Professional”) AND (“Primary Health Care” OR “Primary Healthcare” OR “Primary Care”)
**EMBASE**	('health professionals' OR 'health personnel' OR 'health workforce'/exp) AND ('continuing education'/exp OR 'permanent education') AND ('primary health care' OR 'primary care')
**LILACS**	("permanent education" OR "education continuing") AND ("health professionals" OR "health care professionals" OR "health personnel" OR "wealth workforce") AND ("primary healthcare" OR "primary care")
**GOOGLE SCHOOL**	((permanent education) OR (education continuing)) AND ((health professionals) OR (health care professionals) OR (health personnel)) AND ((primary health care) OR (primary healthcare) OR (primary care))
